# Pliocene oceanic seaways and global climate

**DOI:** 10.1038/srep39842

**Published:** 2017-01-05

**Authors:** Cyrus Karas, Dirk Nürnberg, André Bahr, Jeroen Groeneveld, Jens O. Herrle, Ralf Tiedemann, Peter B. deMenocal

**Affiliations:** 1 Goethe-University Frankfurt, Altenhoeferallee 1, 60438, Frankfurt am Main, Germany; 2 Biodiversity and Climate Research Centre (BIK-F), Senckenberganlage 25, 60325 Frankfurt am Main, Germany; 3 GEOMAR Helmholtz Centre for Ocean Research Kiel, Wischhofstrasse 1-3, 24148 Kiel, Germany; 4 Lamont Doherty Earth Observatory, Palisades, NY 10964, USA; 5 Ruprecht-Karls-Universität Heidelberg, Im Neuenheimer Feld 234, 69120 Heidelberg, Germany; 6 Center for Marine Environmental Sciences (MARUM), University of Bremen, Klagenfurter Strasse, 28359 Bremen, Germany; 7 Alfred Wegener Institute for Polar and Marine Research, Am Alten Hafen 26, 27568 Bremerhaven, Germany

## Abstract

Tectonically induced changes in oceanic seaways had profound effects on global and regional climate during the Late Neogene. The constriction of the Central American Seaway reached a critical threshold during the early Pliocene ~4.8–4 million years (Ma) ago. Model simulations indicate the strengthening of the Atlantic Meridional Overturning Circulation (AMOC) with a signature warming response in the Northern Hemisphere and cooling in the Southern Hemisphere. Subsequently, between ~4–3 Ma, the constriction of the Indonesian Seaway impacted regional climate and might have accelerated the Northern Hemisphere Glaciation. We here present Pliocene Atlantic interhemispheric sea surface temperature and salinity gradients (deduced from foraminiferal Mg/Ca and stable oxygen isotopes, δ^18^O) in combination with a recently published benthic stable carbon isotope (δ^13^C) record from the southernmost extent of North Atlantic Deep Water to reconstruct gateway-related changes in the AMOC mode. After an early reduction of the AMOC at ~5.3 Ma, we show in agreement with model simulations of the impacts of Central American Seaway closure a strengthened AMOC with a global climate signature. During ~3.8–3 Ma, we suggest a weakening of the AMOC in line with the global cooling trend, with possible contributions from the constriction of the Indonesian Seaway.

The tectonic closure history of the Central American Seaway (CAS) is complex and a long lasting process that started during the latest Oligocene and early Miocene^1^. Recent tectonic evidence from land suggests that deep and intermediate connections between both ocean basins were already closed during the middle Miocene^2^. However, manifold evidence from ocean records indicate the existence of shallow (≤200 m) connections between both ocean basins until the Pliocene and that the closure of these were sufficient to affect global climate^3^,^4^,^5^,^6^,^7^,^8^,^9^,^10^,^11^. Most notably, paleoceanographic data from either side of the Panamanian land bridge indicate that the further restriction of the CAS reached a critical threshold between ~4.8–4 Ma with marked impacts on ocean circulation and global climate^5^,^6^,^7^,^8^. Climate model simulations of this closure indicate a significant increase of the AMOC leading to higher sea surface temperatures (SST) and sea surface salinities (SSS) in the Northern Atlantic^7^,^9^,^12^. In contrast, the Southern Hemisphere experienced cooling and freshening through the increased transport of warmth to the Northern Atlantic, a climatic consequence known as “heat piracy”. Following CAS closure, the restriction of the Indonesian Seaway between ~4 and 3 Ma played a prominent role in changing ocean currents and climate in the tropical eastern Indian Ocean and southwest Pacific Ocean^13^,^14^,^15^,^16^ such as the onset of aridity in northwestern Australia^15^. In particular, this tectonic re-organisation of the Indonesian region caused a change in throughflow from warm and salty South Pacific to fresher and cooler North Pacific subsurface water masses with most prominent changes during 3.5–2.95 Ma^13^,^16^. The constriction of the Indonesian Seaway has been suggested to precondition the onset of Northern Hemisphere Glaciation^16^,^17^. Other tectonic-induced closures and openings discussed here include the end of the Messinian Salinity Crisis during the latest Miocene (5.96–5.33 Ma) when the Mediterranean Sea was periodically blocked and connected again to the Atlantic Ocean with simulated effects on the AMOC^18^,^19^. Also, we discuss possible climatic effects due to the opening of the Bering Strait that is assumed of having taken place during the Pliocene and proposed to be important for North Atlantic SST and the AMOC strength^20^,^21^,^22^.

In this study, we focus on Pliocene climatic changes on the ocean surface deciphered from proxy records of the South Atlantic Deep Sea Drilling Project (DSDP) Site 516A (30°17′S; 35°17′W) and of North Atlantic DSDP Site 552A (56°02′N23°13′W; Fig. 1). Our strategy is based on data and model simulations showing that the sea surface at the selected core locations likely reacted sensitively on past changes in AMOC in general^23^,^24^,^25^,^26^,^27^,^28^ and in particular in response to the constriction/closure of the CAS^7^,^9^,^12^. Site 516A lies within the Subtropical Gyre at the edge of the Brazil Current, which constitutes the South Atlantic Current and is suitable to monitor SST and SSS signature responses in the Southern Hemisphere due to changes in AMOC strength. For instance, a weakening of the AMOC should have resulted in the strengthening of the warm Brazil Current leading to warmer temperatures and increasing salinities^23^,^24^. North Atlantic Site 552A is located within the influence of the warm and saline North Atlantic Current at the surface layer, which transports heat and salt towards the northern North Atlantic^27^ and is therefore well situated to monitor SST and SSS changes in this northern limb of the AMOC^7^,^9^,^12^,^25^,^26^,^27^. Accordingly, changes in the strength of the AMOC are likely reflected in the interhemispheric SST difference between sites 552A and 516A and hence, can be used for assessing the strength of the AMOC^25^,^28^. The underlying assumption is that changes in the volume flux of warm waters towards the North Atlantic are directly related to North and South Atlantic SST^25^,^28^. We followed this approach using our SST and SSS reconstructions in combination with benthic δ^13^C values from the South Atlantic Site 1264 (smoothed record; ref. 29), which are indicative of changes in North Atlantic Deep Water strength^29^,^30^. North Atlantic Deep Water represents the deep-water return route of the shallow warm water transport towards the North Atlantic. Hence, we here monitor changes in the entire AMOC – including the shallow and the deep circulation. Paired Mg/Ca and δ^18^O measurements of the planktic foraminifera *Globigerinoides sacculifer* and *Globigerina bulloides* were used to reconstruct SST_Mg/Ca_ and changes in relative SSS expressed as δ^18^O_seawater_ values (Supplementary Information). We examine long-term trends, as well as interhemispheric and interbasinal gradients in SST_Mg/Ca_ and δ^18^O_seawater_ (expressed as smoothed records) to explore long-term supra-regional oceanographic and climate variability responding to tectonic changes. For consistent age control, we established benthic δ^18^O stratigraphies for sites 552A and 516A (Supplementary Information).

## Results and Discussion

**Figure 2:**
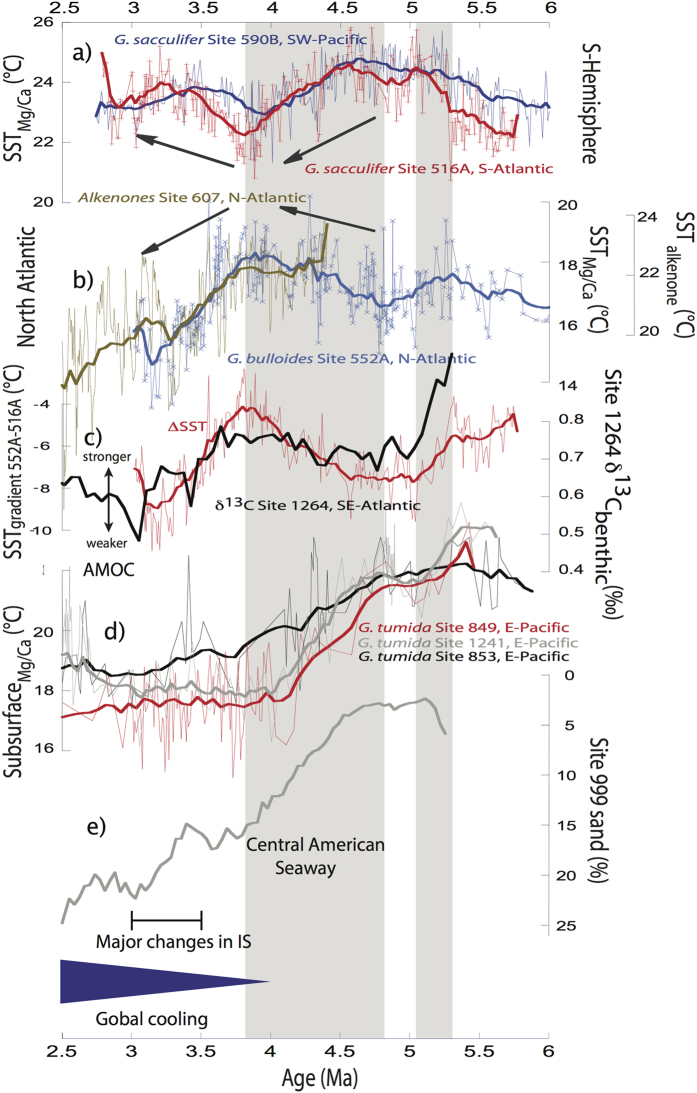
Pliocene paleoceanographic changes. (**a**) *G. sacculifer* SST_Mg/Ca_ records from Southern Hemisphere sites 516A (red line) and 590B^14^ (blue line). (**b**) *G. bulloides* SST_Mg/Ca_ record from Site 552A (blue line), and alkenone derived SST from Site 607 (ref. 42) from the North Atlantic (brown line). (**c**) Interhemispheric SST_Mg/Ca_ gradient between sites 552A and 516A (red; interpreted as deviation from 0) and benthic δ^13^C record from Site 1264 (ref. 29; smoothed black line). (**d**) *G. tumida* subsurface Mg/Ca derived temperatures from Site 1241 (ref. 8; green line), and other sites 848 (blue line), 849 (red line), and 853 (black line) from ref. 32. (**e**) Sand percentages at Site 999 (ref. 5; smoothed line). Shaded areas indicate distinct changes in proxy records due to the tectonic constrictions of the CAS and the Mediterranean Seaway IS = Indonesian Seaway. Thick lines represent smoothed lines based on a Stineman function with ±10% data range (performed with Kaleidagraph 4.1).

**Figure 3:**
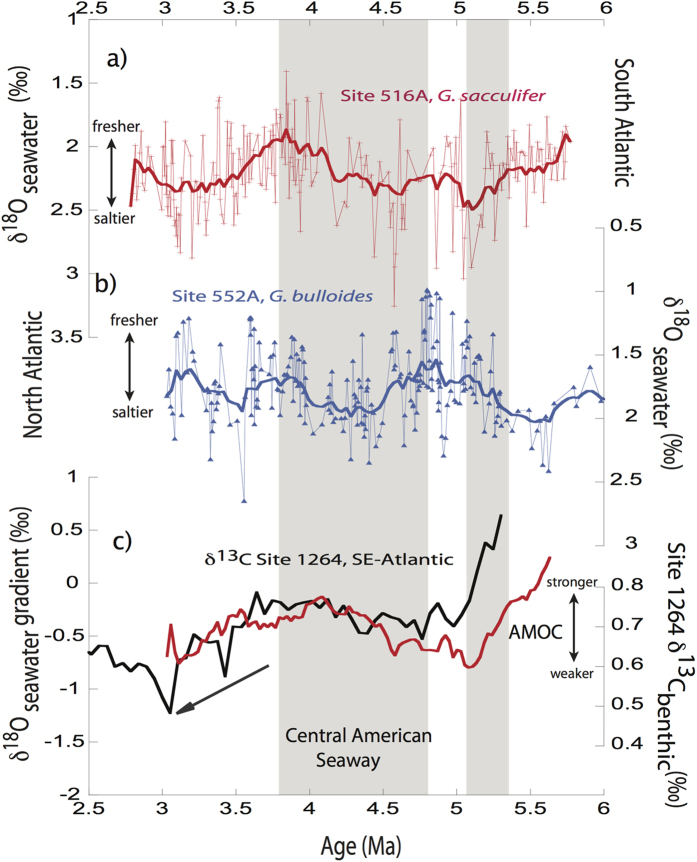
Surface salinity reconstructions during the Pliocene. (**a**) δ^18^O_seawater_ record from South Atlantic Site 516A (red line). (**b**) North Atlantic Site 552A δ^18^O_seawater_ record (blue line). (**c**) δ^18^O_seawater_ gradient between sites 552A and 516A as indication of relative changes of ancient salinities (red; interpreted as deviation from 0) and benthic δ^13^C record from Site 1264 (ref. 29; smoothed black line). Thick lines represent smoothed lines based on a Stineman function with ±10% data range (performed with Kaleidagraph 4.1).

North Atlantic DSDP Site 552A shows an inverse SST_Mg/Ca_ development compared to South Atlantic Site 516A and Southwest Pacific Site 590B^14^ during the entire time period studied, suggesting that a long-term interhemispheric seesaw might have existed in the Atlantic Ocean at least since the latest Miocene (Fig. 2a,b). This tight anti-correlation between records, indeed, encourages us to use the Pliocene SST_Mg/Ca_ gradient between North Atlantic Site 552A and South Atlantic Site 516A as a reflection of AMOC variability^25^,^28^. Robust independent support for our approach^25^,^28^ stems from a recent benthic δ^13^C record from Site 1264 in the Southeast Atlantic (Fig. 2c; ref. 29). This site location is bathed in the North Atlantic Deep Water and the δ^13^C record has been shown to be very sensitive to changes in North Atlantic Deep Water export into the South Atlantic^30^. The close similarity between the interhemispheric SST_Mg/Ca_ gradient between sites 552A and 516A and the benthic δ^13^C record from the Southeast Atlantic Site 1264 (ref. 29; lower δ^13^C values mean less influence from North Atlantic Deep Water^29^,^30^) impressively support the tight connection of North Atlantic Deep Water formation and the Atlantic interhemispheric temperature gradient^25^,^28^ (Fig. 2c). This tight relationship is also reflected in our calculated δ^18^O_seawater_ gradient pointing to an enhanced interhemispheric SSS gradient with relative freshening at North Atlantic Site 552A and more saline conditions at South Atlantic Site 516 at times of AMOC weakening (Fig. 3a, b,c; refs 9, 12 and 23). Together, both our SST_Mg/Ca_ and δ^18^O_seawater_ gradients reacted sensitively due to changes in AMOC.

We note an increasing SST_Mg/Ca_ gradient between the North and South Atlantic of ~3 °C during ~5.6–5 Ma with most distinct cooling of the North Atlantic Site 552A (~1.5 °C) and warming of the South Atlantic Site 516A (~2 °C; Fig. 2a,b,c) at ~5.3 Ma. This event appears synchronous to the Messinian Salinity Crisis. During this time the Mediterranean Sea was periodically blocked from and connected again with the North Atlantic^18^ with effects on the salinity of Mediterranean Outflow Water^19^. A recent modelling study^19^ suggested that a related change from more to less saline Mediterranean Outflow Water conditions would have caused a significant weakening of the AMOC in line with a typical bipolar temperature and salinity asymmetry. Our observed pattern of an enlarged bipolar SST_Mg/Ca_ gradient in the Atlantic Ocean during 5.6–5 Ma is synchronous to the evolving interhemispheric SSS gradient with a mean amplitude of ~0.5‰ (Figs 2c and 3c). Jointly, with the significant decrease of ~0.3‰ of the benthic δ^13^C record from the Southeast Atlantic Site 1264 at ~5.3 Ma^29^, these changes in the proxy records indicate a substantial weakening of the AMOC (Figs 2c and 3c).

From ~4.8–4 Ma, a strong SSS gradient developed between Caribbean Site 999 and tropical eastern Pacific Ocean Site 851 Ma^6^,^31^, while the eastern Pacific subsurface temperatures cooled and accordingly, the thermocline shoaled^7^,^8^,^32^,^33^,^34^ (Fig. 2d). At the same time, a continuous amplification of the AMOC was proposed^5^, witnessed by increasing sand percentages at Caribbean Site 999 (Fig. 2e; ref. 5). These were interpreted in terms of increasing carbonate preservation and hence, the presence of less corrosive, well-oxygenated North Atlantic Deep Water^5^. The benthic δ^13^C gradient between Caribbean sites 1000 and 925 also suggests the strengthening of the AMOC with better-ventilated Upper North Atlantic Deep Water^7^. All these developments are evident for the continuous constriction of the CAS, the climatically relevant effects of which include the strengthened transport of warmer, saltier North Atlantic Current waters towards the northern North Atlantic^5^,^6^,^7^,^8^,^9^,^12^. According to a multi model simulation^9^, the constriction of the CAS during the early Pliocene should have led to both: The shoaling of the thermocline in the tropical eastern Pacific and an interhemispheric “seesaw pattern” in SST and SSS. In consequence, the North Atlantic should have warmed with more saline conditions and the South Atlantic including the Southern Ocean should have freshened and cooled^9^.

However, a recent study based on benthic δ^13^C records from the deep North and South Atlantic^30^ suggested that during the early Pliocene only the production of upper North Atlantic Deep Water was increased due to the constriction of the CAS with minuscule climatic effects^30^.

**Figure 4:**
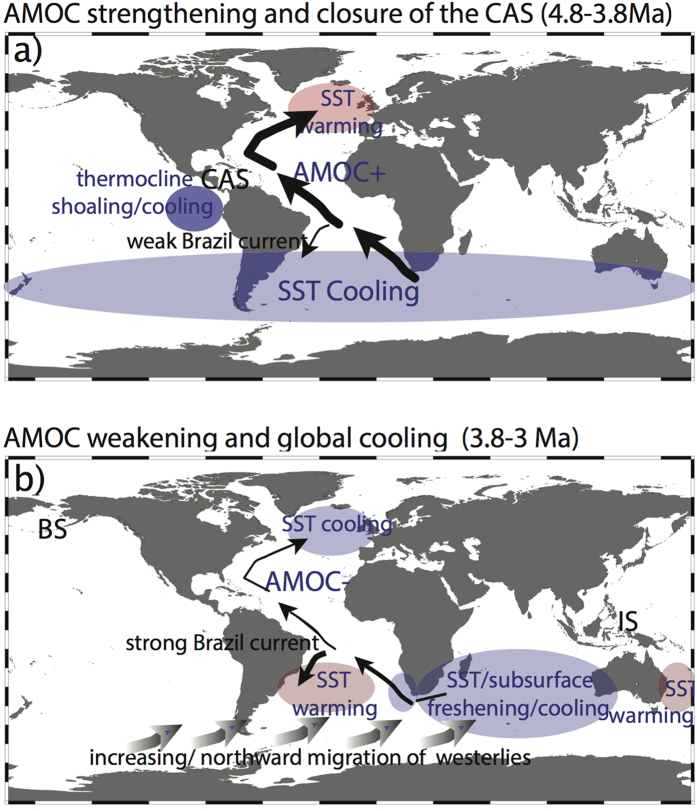
Summary charts showing AMOC-related climatic changes during the constriction of the CAS. (**a**) and global cooling (**b**) respectively. Oceanographic changes as changes in AMOC variability, changes in temperatures and ocean currents are schematically indicated and based on the results of this study and other studies^5^,^6^,^7^,^8^,^9^,^12^,^13^,^14^,^15^,^16^,^17^,^29^,^32^,^43^,^44^,^45^,^46^,^50^,^51^,^52^. IS = Indonesian Seaway, BS = Bering Strait. Charts were created with Ocean Data View (http://odv.awi.de; version 4.5.1)^55^.

Our proxy data are largely consistent with the model results and data studies suggesting significant climatic changes due to the constriction of the CAS^5^,^6^,^7^,^8^,^9^,^12^,^22^ (Fig. 4a). During ~4.8–3.8 Ma, synchronous sea surface cooling of ~2 °C at Southern Hemisphere sites 516 A and 590B^14^ and sea surface warming by ~2 °C in the North Atlantic (Site 552A) generate a diminishing interhemispheric SST gradient (~3.5 °C) between North and South Atlantic sites 552A and 516A (Fig. 2a,b,c). In accordance with the slightly increasing δ^13^C values at South Atlantic Site 1264 (ref. 29), this decreasing temperature gradient supports the amplification of the AMOC at ~4.8–3.8 Ma^5^,^7^,^9^,^25^ (Fig. 2c). Our δ^18^O_seawater_ values further imply the notion of significant climatic changes and a stronger AMOC during this time^9^ (Fig. 3c): During ~4.8–3.8 Ma, the δ^18^O_seawater_ gradient decreased between the North and the South Atlantic of ~0.5‰ suggesting more saline conditions at North Atlantic Site 552A.

We note, however, in accordance to Bell *et al*. (ref. 30) that during ~4.8–3.8 Ma AMOC strengthening was better developed in the upper AMOC branch than in the deep AMOC as seen at Southeast Atlantic Site 1264 (ref. 29). Stronger circulation in the upper AMOC branch is supported by the distinctly increased sand percentages at Caribbean Site 999 (ref. 5; Fig. 2e) and the enlarged benthic δ^13^C gradient between Caribbean sites 1000 and 925 (ref. 7).

Our proxy data from both hemispheres support the hypothesis that the CAS constriction and the closely related strengthening of the AMOC even affected the tropical East Pacific by cooling/shoaling of the thermocline during ~4.8–4 Ma^7^,^9^ (Fig. 2d). Cessi *et al*. (ref. 35) proposed that the amplification of the AMOC, which is part of the globally spanning ocean circulation conveyor enhanced the heat transport from the tropical Pacific towards the North Atlantic. In consequence, the North Atlantic warmed, while the tropical Pacific thermocline cooled and shoaled^35^. The latter process was even fostered by the cooling of the Southern Ocean as evidenced by our sites 516A and 590B^14^ SST_Mg/Ca_ records. The related intensified formation, northward spread, and equatorial upwelling of southern-sourced mode and intermediate waters due to strengthened wind circulation fostered global cooling through ocean-atmosphere processes^36^ and should have considerably contributed to the shoaling and cooling of the tropical eastern Pacific thermocline^7^,^32^,^37^. This process apparently started regionally as early as ~4.4 Ma, marked by the increasing SST_Mg/Ca_ gradient between the equatorial West Pacific Site 806 and the East Pacific Site 846^38^,^39^.

Synchronously with the subsequent global cooling trend^40^,^41^, we observe during ~3.8–3 Ma a cooling of ~2–3 °C at the North Atlantic sites 552A and 607 (ref. 42) and a warming at South Atlantic Site 516A of the same magnitude leading to an increased interhemispheric temperature gradient between the North and South Atlantic of ~4 °C (less pronounced at Southwest Pacific Site 590B^14^) (Fig. 2a,b,c). Also, the δ^18^O_seawater_ gradient between the North and South Atlantic increased by ~0.5‰ with a relative freshening of North Atlantic Site 552A (Fig. 3c). Synchronously, benthic δ^13^C values at Site 1264 (ref. 29) decreased by ~0.25‰ indicative of a weaker North Atlantic Deep Water export into the South Atlantic (Figs 2c and 3c). All these proxy data suggest a weakening of the AMOC^25^,^26^,^28^ that is further supported by a comparison of Nd and Pb isotopes from the South and North Atlantic43^43^. Between ~4 and ~3 Ma both isotope signatures diverged between the North and the South Atlantic pointing to a weakened North Atlantic Deep Water circulation^43^.

Driving mechanisms for the Pliocene weakening of the AMOC are complex and include climatic changes in the North Atlantic and/or the Southern Hemisphere and might be a combination of the global cooling trend and/or tectonic changes in the Indonesian region or even the Bering Strait (Fig. 4b). For instance, as Pliocene cooling was more pronounced at high latitudes^41^ cooling of the Southern Hemisphere high latitudes would have initiated the northward displacement of the westerly wind belts which in turn, would have substantially weakened the AMOC^44^ (Fig. 4b).

The constriction of the Indonesian Seaway with the related emergence of the Maritime Continent with a gain in landmasses of ~60% including many islands contributed to the general global cooling trend since ~5 Ma^45^. Intensified weathering of basaltic rocks along with the rising of the Maritime Continent might have contributed to a long-term CO_2_ drawdown^45^. Further, the slight tectonic-induced decrease of eastern tropical Pacific SST might have led to a stronger east-west Pacific SST gradient causing a stronger Walker circulation with far reaching climatic effects on North America^45^. The tectonic-induced distinct freshening of the subsurface eastern tropical Indian Ocean due to the restriction of the Indonesian Throughflow waters during ~3.5–2.95 Ma^13^,^15^ (Fig. 4b) might have affected the Agulhas Current via the “warm water” route^13^,^46^. Freshening at the subsurface level in this current system was simulated to result in a substantial weakening of the AMOC^47^. Other temperature and salinity reconstructions along the “warm water route” in the South Atlantic also witnessed the climatic effects of the constriction of the Indonesian Seaway in combination with global cooling showing cooling and/or freshening since ~4 Ma (sites 1090, 1264 and 1084; refs 48, 49 and 17). Model simulations^50^,^51^,^52^ proposed the reduced poleward heat flux resulting from the constriction/closure of the Indonesian Seaway including the cooling of the Leeuwin Current with dramatic consequences for the climate of western Australia (drying), and a cooling of the southern South Atlantic and the Benguela upwelling region (Site 1084; ref. 17), respectively. This was largely confirmed by proxy data in those ocean regions^15^,^17^.

During the same time at ~3.6 Ma, a significant change in the migration pattern of Pacific molluscs into the Atlantic Ocean evidenced the opening of the Bering Strait^21^. This opening seaway was simulated to significantly lower SST in the North Atlantic and weaken the AMOC circulation^20^,^22^. However, the exact timing of this event was recently questioned and estimates now range from the early Pliocene until the late Pliocene^21^,^22^,^53^.

## Conclusions

We show that the long-term Pliocene changes in the Atlantic interhemispheric temperature and δ^18^O_seawater_ gradients presented here reacted synchronously with a published benthic δ^13^C record from the Southeast Atlantic indicative of changes in North Atlantic Deep Water^29^,^30^. This similarity supports the hypothesis that the SST and SSS gradients between the North and South Atlantic closely reflect AMOC changes^25^,^26^,^28^. Overall, the proxy data allow to test the impacts of tectonic reorganisations of ocean gateways on the Pliocene climate. We suggest an early reduction of the AMOC at ~5.3 Ma, possibly related to the end of the Messinian Salinity Crisis. Between ~4.8–3.8 Ma, the reduced SST_Mg/Ca_ and δ^18^O_seawater_ gradients between the North and the South Atlantic support hypotheses claiming that the CAS closure strengthened AMOC with prominent climatic effects on both hemispheres^5^,^6^,^7^,^8^,^9^,^12^,^22^ (Fig. 4a). During ~3.8–3 Ma, our surface proxy data in combination with the benthic δ^13^C record from the Southeast Atlantic^29^ suggest the weakening of the AMOC (Fig. 4b) that might be a complex climatic effect of global cooling possibly supported by tectonic changes in the Indonesian region (Fig. 4b).

## Data Availability

Data of this study are available electronically at the World Data Center for Paleoclimatology (WDC Paleo), https://www.ncdc.noaa.gov/paleo/wdc-paleo.html”.

## Additional Information

**How to cite this article**: Karas, C. *et al*. Pliocene oceanic seaways and global climate. *Sci. Rep.* **7**, 39842; doi: 10.1038/srep39842 (2017).

**Publisher's note:** Springer Nature remains neutral with regard to jurisdictional claims in published maps and institutional affiliations.

## Supplementary Material

Supplementary Information

## Figures and Tables

**Figure 1:**
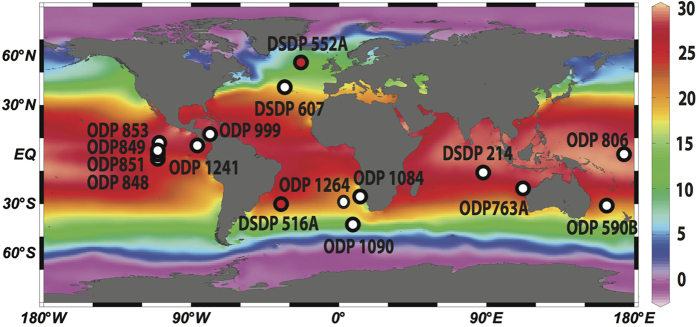
Modern annual sea surface temperature distribution at 30 m water depth^54^. ODP/DSDP sites are indicated. Paleoceanographic proxy data were generated for South Atlantic DSDP Site 516A and North Atlantic Site 552A (red dots). Chart was created with Ocean Data View (http://odv.awi.de; version 4.5.1)^55^.
