# Supplementary material for: Pliocene oceanic seaways and global climate
Source: Sci Rep. 2017 Jan 5;7:39842. doi: 10.1038/srep39842 (PMC5215318; doi:10.1038/srep39842)
Supplement: Supplementary Information [file srep39842-s1.pdf]

Supplementary Information

Pliocene oceanic seaways and global climate

Cyrus Karas[1,2,3,4]*, Dirk Nürnberg[3], André Bahr[5], Jeroen Groeneveld[6], Jens O. Herrle[1,2], Ralf Tiedemann[7], Peter B. deMenocal[4]

[1] Goethe-University Frankfurt, Altenhoeferallee 1, 60438, Frankfurt am Main, Germany
[2] Biodiversity and Climate Research Centre (BIK-F), Senckenberganlage 25, 60325 Frankfurt am Main, Germany
[3] GEOMAR Helmholtz Centre for Ocean Research Kiel, Wischhofstrasse 1-3, 24148 Kiel, Germany
[4] Lamont Doherty Earth Observatory, Palisades, NY 10964, USA
[5] Ruprecht-Karls-Universität Heidelberg, Im Neuenheimer Feld 234, 69120 Heidelberg, Germany
[6] Center for Marine Environmental Sciences (MARUM), University of Bremen, Klagenfurter Strasse, 28359 Bremen, Germany
[7] Alfred Wegener Institute for Polar and Marine Research, Am Alten Hafen 26, 27568 Bremerhaven, Germany

Analysis of foraminiferal $\delta^{18}O$ and Mg/Ca

For stable oxygen isotope ($\delta^{18}O$) and Mg/Ca analyses we selected ~20-30 specimens of shallow dwelling *Globigerinoides sacculifer (*without sac-like chamber) from Deep Sea Drilling Project (DSDP) Site 516A and ~30-40 specimens of shallow dwelling planktonic foraminifera *Globigerinoides bulloides* from DSDP Site 552A. For benthic isotope stratigraphy of both cores we selected 1-4 specimens of C*ibicidoides wuellerstorfi*. The selected planktonic foraminiferal species *G. bulloides* and *G. sacculifer* are proven faithful recorders of surface ocean conditions during the Pliocene[1, 2, 3]. Both foraminiferal species live in the upper ~50 m water depth[4, 5] and are suggested to record annual ocean conditions (refs. 6, 2 and references therein). In order to gain enough material for analyses and to avoid size effects in $\delta^{18}O$ values and Mg/Ca[7] tests of *G. bulloides* were selected from the narrow 215-315 μm size fraction, while tests of *G. sacculifer* were selected from the 315-355 μm size fraction. Foraminiferal tests were gently crushed, mixed and split into two thirds used for

Mg/Ca- analyses, and one third for stable isotope measurements. Isotope measurements were either conducted on a Thermo Scientific MAT-253 mass spectrometer equipped with a CARBO Kiel IV device and on a Thermo Scientific MAT-253 equipped with a Gas Bench II. Analytical precession was better than $\pm$ 0.08 ‰ for $\delta^{18}O$; $\pm \sigma$. All values are reported relative to Vienna Pee Dee Belemnite (VPDB, based on calibration directly to National Bureau of Standards (NBS-19).

For Mg/Ca analyses, samples were cleaned according to an established cleaning protocol[8] (non reductive). In order to minimize possible contamination of the measured Mg/Ca ratios with clays or Mn carbonates the sample solutions were centrifuged for 2.5 min (10000 rpm) before measurement. The residual of 50 µL were discarded. Solutes used for analyses contained 1 ppm Yttrium as an internal standard. Measurements were conducted on an iCAP 6000 (Thermo Scientific™) ICP-OES with a combined radial and axial optical configuration. Spectral lines used were 315.8 nm for Ca (radial) and 280.2 nm for Mg (axial), normalized to the 371.0 nm (Ca) and 320.3 nm (Mg) lines of Yttrium, respectively. Calibration was done following an intensity ratio calibration approach[9]. Results were standardized and trend-corrected to the ECRM 752-1 standard with a reference value of 3.762 mmol/mol for Mg/Ca[10]. The analytical error for the Mg/Ca ratios was ~0.3%. Replicate analyses of a few aliquots of crushed samples showed a standard deviation of ~0.1 mol/mol (rel. std. dev. ≤3%). To monitor contamination we additionally measured Fe (238.2 nm, axial) and Mn (257.6 nm, axial). After cleaning, Fe/Ca values of samples from core site 516A were generally below ~0.1 mmol/mol and those from core Site 552A mainly below ~0.18 mmol/mol indicating no significant contamination with clays. Also, monitoring of Mn/Ca ratios at Site 516A (mean of ~0.08 mmol/mol) and at Site 552A (mean of ~0.19 mmol/mol) showed no indication for contamination with Mn-carbonates. Mg/Ca ratios of *G. bulloides* were translated into temperatures by using the multispecies calibration[11]: Mg/Ca = 0.52 (±0.0085) exp (0.10 x SST). In order to stay consistent with published *G. sacculifer* $SST_{Mg/Ca}$ from the southwest

Pacific Site 590B[12] used for comparison we applied the multispecies calibration[5]: Mg/Ca = 0.38 (±0.02) exp (0.09 (±0.003) x SST) to convert *G. sacculifer* Mg/Ca ratios into temperatures. The absolute error in determining $SST_{Mg/Ca}$ is up to ~ ±1.5 °C[2, 5, 11].

Temporal changes in Mg/Ca of seawater[13, 14] and their effects on absolute foraminiferal $SST_{Mg/Ca}$ are a matter of current research and the necessity of a correction of initial Pliocene foraminiferal Mg/Ca values affords discussion[15, 16]. The main uncertainties in this debate[16] include the lag of an exact knowledge of Pliocene Mg/Ca seawater values and the residence times of Mg and Ca in seawater (14 Ma and 1 Ma, respectively[17]), which make it difficult to assess changes in $Mg/Ca_{seawater}$ on time scales <2 Ma. In addition, possible species-specific differences in the sensitivity to changes in $Mg/Ca_{seawater}$ afford consideration.

Irrespective of these considerations, absolute $SST_{Mg/Ca}$ estimates for the Pliocene might have been higher than reported here when taking into account both published non-linear Mg/Ca_{foram}-$Mg/Ca_{seawater}$ relationships, and a lowering of the Mg/Ca-temperature sensitivity at lower than modern $Mg/Ca_{seawater}$[15]. If applying the Evans et al.[15] $Mg/Ca_{seawater}$ correction (based on *G. ruber*) for a lowered $Mg/Ca_{seawater}$ value of ~4.3 mol/mol between 3.0-5.0 Ma (modern value: 5.2 mol/mol), absolute Pliocene $SST_{Mg/Ca}$ records would increase by about ~1.5-1.7°C[15]. We decided here to report uncorrected $SST_{Mg/Ca}$ as the application of this correction is in the range of the reported absolute error in determining $SST_{Mg/Ca}$ (see above) and would not change our observed relative Pliocene changes, on which our study focuses on.

Preservation of foraminiferal tests

It is well known that calcite dissolution is critical for foraminiferal Mg/Ca, and selective Mg/Ca^{2+}-ion removal starts well above the lysocline[18]. We therefore selected our core sites from well above the critical $\Delta[CO_3^{2-}]$ value of ~21 (±6.6) µmol/kg $\Delta[CO_3^{2-}]$, which was defined as a threshold value of ocean calcite saturation below which the foraminiferal Mg/Ca signal is significantly perturbed[18]. Notably, as sites 552A and 516A are ~3.5 km and ~2.5-3

km shallower than this threshold[18], we assess modern calcite dissolution processes on foraminiferal Mg/Ca negligible, although these considerations only hold for present-day. In order to evaluate the dissolution impact on foraminiferal Mg/Ca through time, the loss of bulk foraminiferal calcite is taken as a sign of calcite dissolution[19, 20]: The higher the loss in calcite, the lower the foraminiferal Mg/Ca. We accomplished weight measurements of foraminiferal tests of *G. bulloides* (Site 552A) and *G. sacculifer* (Site 516A) at low-resolution during the critical time period (~5-3.5 Ma) of severe $SST_{Mg/Ca}$ changes (max. 5°C amplitudes at both sites; S-Fig. 1a, b). 30-35 tests were selected from the same size fraction the Mg/Ca-analyses were performed on. Single average foraminiferal test weights change from 23-33 µg for *G. sacculifer* and 11-16 µg for *G. bulloides*. The test weight records from both sites show a good inverse correlation to the $SST_{Mg/Ca}$ records, with higher weights during periods of lower temperatures (S-Fig. 1a, b). We take this anti-correlation as evidence that our $SST_{Mg/Ca}$ records were not affected by calcite dissolution processes throughout the Pliocene. Rather, it might be speculated that the test weights might have been controlled by changing surface ocean processes. Recent studies suggested a relationship of test weights and surface water [CO_3^{2-}] conditions[21, 22], as well as a study[22] observed a correlation of test weights and $SST_{Mg/Ca}$ during the last 22kyrs due to changes in surface water [CO_3^{2-}] conditions. This relationship might also apply during the Pliocene at our sites.

Age models

We used the initial mbsf scale (meter below sea surface) for establishing the age models for our sites as there were no composite depth records available. That means that e.g. coring gaps, overcoring and disturbing of the upper sediment (up to ~2 m equivalent to ~0.2 Ma) might occur in the beginning of each core section[23]. Hence, we decided to reject those samples from

the beginning of each core section in case they overlapped with the preceding core depths in order to gain a continuous depths record of each site.

Site 516A

For an initial age control we selected nannofossil (depths from ref. 24) and planktic foraminiferal biodatums (depths from www.odsn.de) and updated them according to the ATNTS time scale[25]. For further improvement of the biodatum-based age model, we generated a benthic $\delta^{18}O_{C.\ wuellerstorfi}$ record with an average temporal resolution of ~10 kyrs, which served as a robust basis for the benthic $\delta^{18}O$ stratigraphy (S-Fig. 2a). We tuned the benthic $\delta^{18}O_{C.\ wuellerstorfi}$ record to the global reference LR04 stack[26] using Analyseries 2.0 software[27]. After tuning both $\delta^{18}O$ records correlate well (R= 0.71; S-Fig. 2a). Except for one outlier, the initial nannofossil and planktonic biodatums support our established depth/age relationship with a maximum deviation of ~2.4 meters equivalent to ~0.26 Ma (S-Fig. 2b).

Site 552A

To achieve a robust age model for DSDP Site 552A, we generated a benthic $\delta^{18}O_{C.\ wuellerstorfi}$ record with a temporal resolution of ~9 kyrs for the studied time interval (S-Fig. 3a). We tuned the benthic $\delta^{18}O_{C.\ wuellerstorfi}$ record to the global reference LR04 stack[26] using Analyseries 2.0 software[27]. After tuning, both records show a correlation of R= ~0.6 (S-Fig. 3a). The established depth/age relationship is supported by the depicted magnetostratigraphic[28, 29], nannofossil and foraminiferal biodatums[30] (S-Fig. 3b; depths were taken from refs 28, 29, 30; ages were updated according to ATNTS time scale[25].

Calculation of $\delta^{18}O_{seawater}$

Following previous Pliocene studies[2, 3, 12] we used the combined $\delta^{18}O$ and Mg/Ca-temperature records from the surface dwelling planktonic species *G. sacculifer* and *G. bulloides* to assess past changes in $\delta^{18}O$ of seawater ($\delta^{18}O_{seawater}$), which reliably approximates sea surface salinity (S-Fig. 4). To stay consistent with previous studies[3, 12, 31] we calculated $\delta^{18}O_{seawater}$ using an equation[32], which considers both global ice volume controlled changes and local variations in surface salinities due to regional hydrological changes. As changes in global ice volume equally affect all records studied here and as we are primarily interested in relative changes among site locations, we decided not to correct for changes in global ice volume. A reasonable absolute error in $\delta^{18}O_{seawater}$ values is calculated to $\pm$ 0.3 ‰[33, 34].

Supplementary References:

1. Bartoli, G. et al. Final closure of Panama and the onset of northern hemisphere glaciation. *Earth Planet. Sci. Lett.*, **237**, 33-44 (2005)
2. DeSchepper, S., Head, M. J. & Groeneveld, J. North Atlantic Current variability through marine isotope stage M2 (circa 3.3 Ma) during the mid-Pliocene. *Paleoceanography*, **24**, PA4206, doi:10.1029/2008PA001725 (2009).
3. Karas, C. et al. Mid-Pliocene climate change amplified by a switch in Indonesian subsurface throughflow. *Nature Geoscience* **2**, 434-438, doi: 10.1038/NGEO520 (2009).
4. Schiebel, R., Bijma, J. & Hemleben, C. Population dynamics of the planktic foraminifer Globigerina bulloides from the eastern North Atlantic, *Deep Sea Res*., Part I, **44**, 1701 – 1713, doi:10.1016/S0967-0637(97) 00036-8 (1997).
5. Anand, P., Elderfield, H. & Comte, M. H. Calibration of Mg/Ca thermometry in planktonic foraminifera from a sediment trap time series. *Paleoceanography* **18**, 846, 10.1029/2002PA000846 (2003).
6. Lin, H.-L., Wang, W.-C. & Hung, G.-W. Seasonal variation of planktonic foraminiferal isotopic composition from sediment traps in the South China Sea, *Mar. Micropaleontol.* **53**, 447–460, doi:10.1016/ j.marmicro.2004.08.004 (2004).
7. Elderfield, H., Vautravers, M. & Cooper, M. The relationship between shell size and Mg/Ca, Sr/Ca, d18O, and d13C of species of planktonic foraminifera. *Geochem. Geophys. Geosyst.* **3**, 10.1029/2001GC000194 (2002).
8. Barker, S., Greaves, M. & Elderfield, H. A study of cleaning procedures used for foraminiferal Mg/Ca paleothermometry. *Geochem., Geophys., Geosyst*., **4** (9), 8407, doi:10.1029/2003GC000559 (2003).
9. de Villiers, S., Greaves, M. & Elderfield, H. An intensity ratio calibration method for the accurate determination of Mg/Ca and Sr/Ca of marine carbonates by ICP-AES. *Geochem. Geophys. Geosys.* **3**, doi: 10.1029/2001GC000169. issn: 1525-2027 (2002).

10. Greaves, M. et al. Interlaboratory comparison study of calibration standards for foraminiferal Mg/Ca thermometrie. *Geochem. Geophys. Geosyst.* **9**, Q08010, doi:10.1029/2008GC001974 (2008).
11. Elderfield, H. & Ganssen, G. Past temperature and δ18O of surface ocean waters inferred from foraminiferal Mg/Ca ratios, *Nature* **405**, 442–445 (2000).
12. Karas, C., Nürnberg, D., Tiedemann, R. & Garbe-Schönberg, D. Pliocene climate change of the Southwest Pacific and the impact of ocean gateways. *Earth Planet. Sci. Lett.* **301**, 117-124 (2011).
13. Wilkinson, B. H. & Algeo, T. J. Sedimentary carbonate record of calcium-magnesium cycling. *Am. J. Sci*., doi:10.2475/ajs.289.10.1158 (1989).
14. Stanley, S. & Hardie, L. Secular oscillations in the carbonate mineralogy of reef-building and sediment-producing organisms driven by tectonically forced shifts in seawater chemistry. *Palaeogeogr. Palaeoclimatol. Palaeoecol.* **144**, 3–19 (1998).
15. Evans, D., Brierley, C., Raymo, M.E., Erez, J. & Müller, W. Planktic foraminifera shell chemistry response to seawater chemistry: Pliocene–Pleistocene seawater Mg/Ca, temperature and sea level change. *Earth Planet. Sci. Lett.* **438**, 139-148 (2016).
16. Seki, O. et al. Paleoceanographic changes in the Eastern Equatorial Pacific over the last 10 Myr. *Paleoceanography* **27**, PA3224, doi:10.1029/2011PA002158 (2012).
17. Li, Y.-H. A brief discussion on the mean oceanic residence time of elements. *Geochim. Cosmochim. Acta* **46**, 2671–2675 (1982).
18. Regenberg, M., Regenberg, A., Garbe-Schönberg, D. & Lea, D. W. Global dissolution effects on planktonic foraminiferal Mg/Ca ratios controlled by the calcite-saturation state of bottom waters. *Paleoceanography* **29**, 127-142, doi:10.1002/2013PA002492 (2014).
19. Lohmann, G. P. A model for variation in the chemistry of planktonic foraminifera due to secondary calcification and selective dissolution. *Paleoceanography* **10**, 445-447 (1995).
20. Broecker, W. S. & Clark E. An evaluation of Lohmann's foraminifera weight dissolution index. *Paleoceanography* **16**(5), 531–534 (2001).
21. Lombard, F., da Rocha, R. E., Bijma, J. & Gattuso, J.-P. Effect of carbonate ion concentration and irradiance on calcification in foraminifera. *Biogeosciences* **7**, 247-255 (2010).
22. Naik, S. S., Naidu, P. D., Govil, P. & Godad, S. Relationship between Weights of Planktonic Foraminifer Shell and Surface Water CO_3= Concentration during the Holocene and Last Glacial Period. *Mar. Geol.* **275** (1-4), 278-282 (2010).
23. Ruddiman, W. F., Cameron, D. & Clement, B. M. Sediment disturbance and correlation of offset holes drilled with the hydraulic piston corer: Leg 94. *Initial Rep. Deep Sea Drilling Project* **94**, 615-634 (1987).
24. Berggren, W. A., Aubry, M. P. & Hamilton, N. Neogene magnetobiostratigraphy of Deep Sea Drilling Project Site 516 (Rio Grande Rise, South Atlantic). In Barker, P. F., Carlson, R. L. & Johnson, D. A. et al. Deep Sea Drilling Project, Initial Reports, **72**, 675–713, (U.S. Government Printing Office, Washington D.C., 1983)
25. Lourens, L. J. et al. in Gradstein, F.M., Ogg, J., et al. (Eds.), *A Geologic Time Scale 2004*: Cambridge (Cambridge Univ. Press, 2004), Appendix 2.
26. Lisiecki, L. E. & Raymo, M. E. A Pliocene-Pleistocene stack of 57 globally distributed benthic d18O records. *Paleoceanography* **20**, 1 PA1003, doi:10.1029/2004PA001071 (2005).
27. Paillard, D., Labeyrie, L. & Yiou, P. Macintosh program performs time-series analysis, *Eos Trans. AGU* **77**: 379 (1996).

28. Raymo, M. E., Hodell, D. & Jansen, E. Response of deep ocean circulation to initiation of Northern Hemisphere Glaciation (3-2 Ma). *Paleoceanography* **7**, 645-672.
29. Keigwin, L. D., Aubry, M.-P. & Kent, D. V. North Atlantic late Miocene stable-isotope stratigraphy, biostratigraphy, and magnetostratigraphy. In Ruddiman, W. F. et al., (Eds), Deep Sea Drilling Project, *Initial Reports* **94**, 935–963, (U.S. Government Printing Office, Washington D.C., 1987).
30. Backman, J. et al. Biostratigraphy of Leg 81 Sediments- a high latitude record. In Roberts, D. G., Schnitker, D., et al. (Eds), Deep Sea Drilling Project, *Initial Reports* **81**, 855–860, (U.S. Government Printing Office, Washington D.C., 1984).
31. Karas, C., Nürnberg, D., Tiedemann, R. & Garbe-Schönberg, D. Pliocene Indonesian Throughflow and Leeuwin Current dynamics: Implications for Indian Ocean polar heat flux. *Paleoceanography* **26**, PA2217, doi:10.1029/2010PA001949 (2011).
32. Shackleton, N. J. & Hall, M. A. Attainment of isotope equilibrium between ocean water and the benthonic foraminiferal genus Uvigerina. Isotopic changes in the ocean during the last glacial. *Cent. Nat. Rech. Sci. Colloq. Int.* **219**, 203 (1974).
33. Schmidt, G. Error anaysis of paleosalinity calculations. *Paleoceanography* **14**, 422–429 (1999).
34. Rohling, E. J. Progress in paleosalinity: overview and presentation of a new approach. *Paleoceanography* **22**, PA3215. doi:10.1029/2007PA001437 (2007).

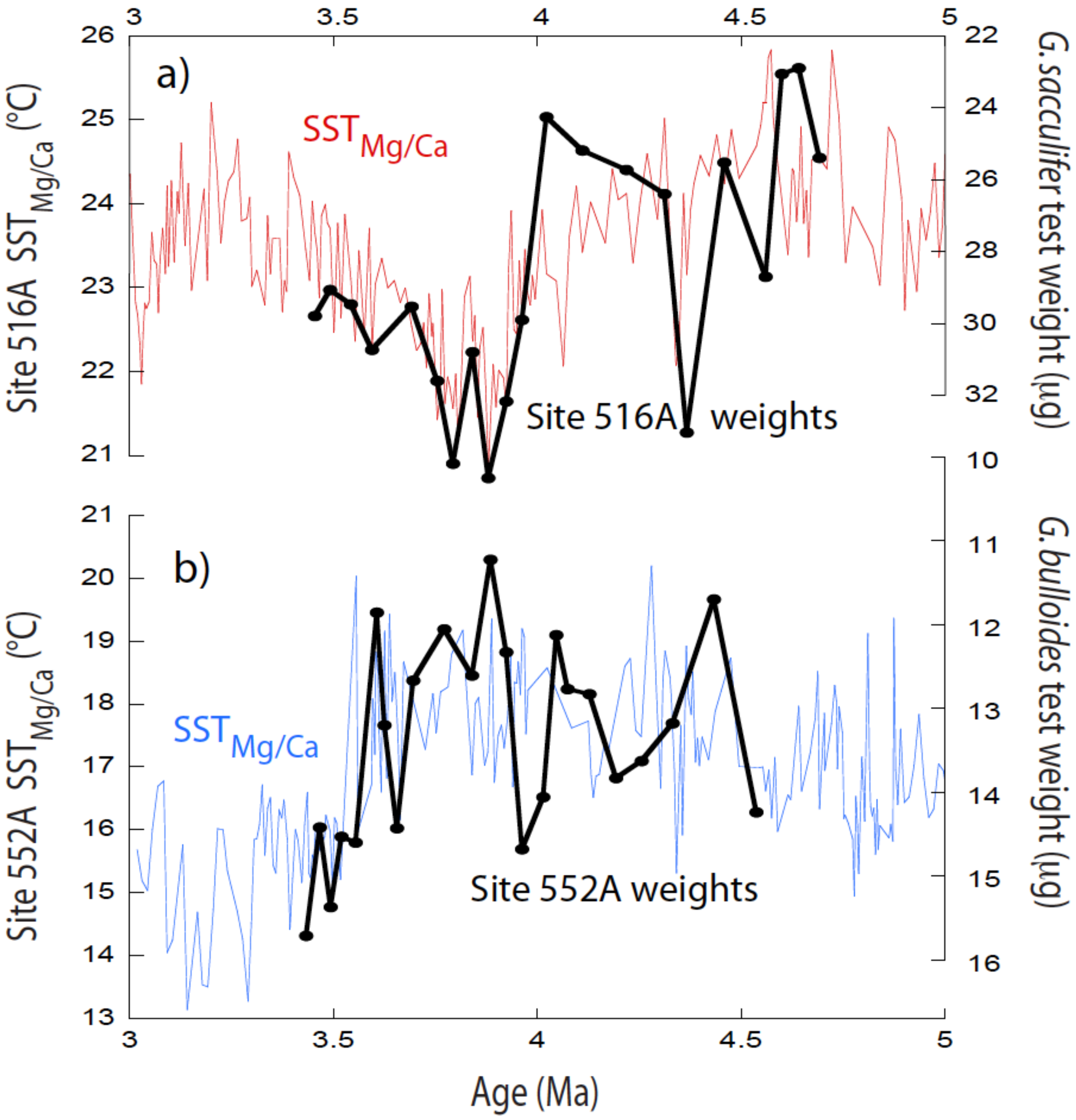

S-Fig. 1: Comparison of $SST_{Mg/Ca}$ and foraminiferal test weights of sites 516A and 552A. (a) Site 516A *G. sacculifer* $SST_{Mg/Ca}$ (red) and G. *sacculifer* test weights (black). (b) Site 552A *G. bulloides* $SST_{Mg/Ca}$ (red) and *G. bulloides* test weights (black). Note that test weights are plotted inversely.

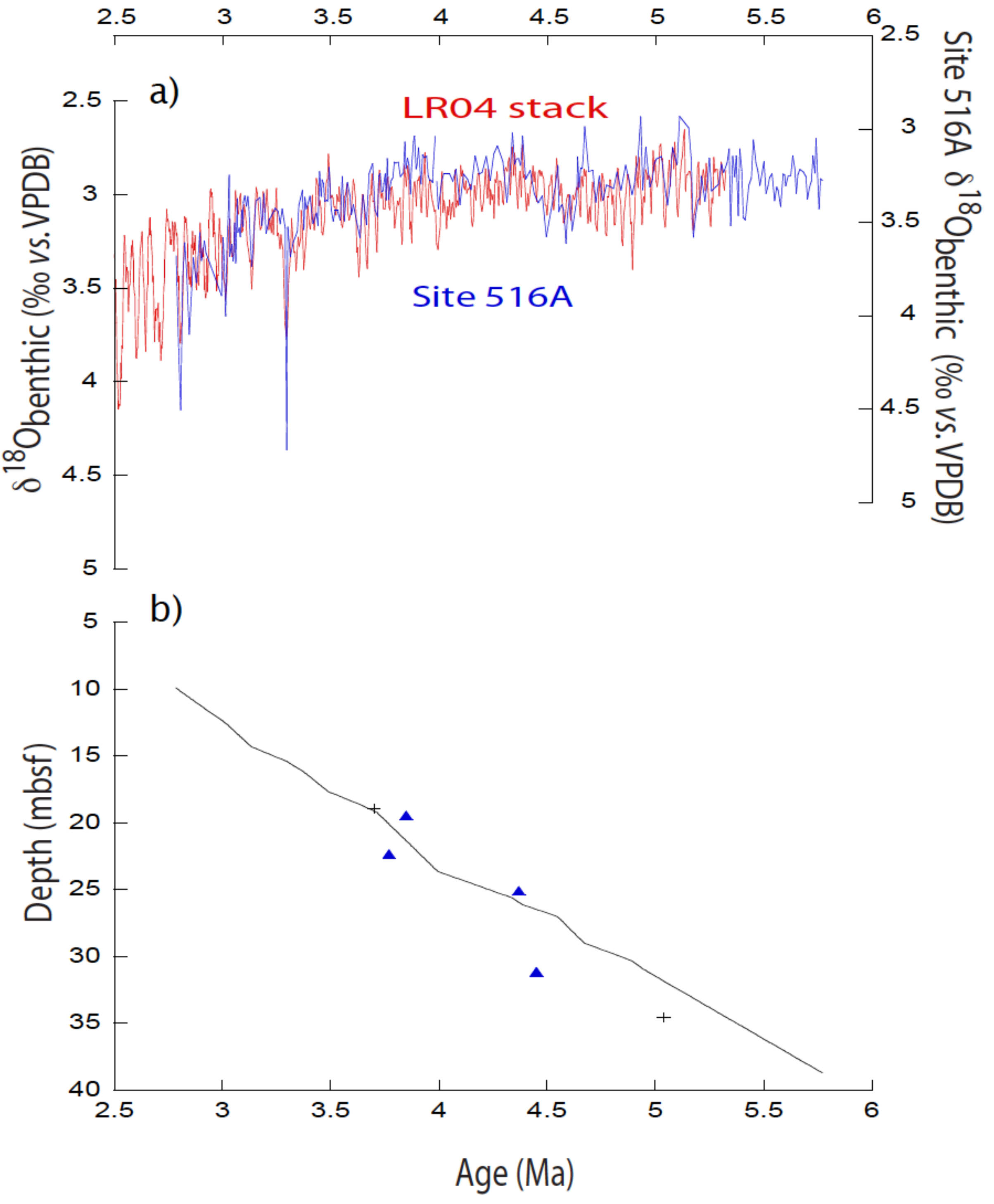

S-Fig. 2: Age model for Site 516A. (a) Tuning of the benthic $\delta^{18}O_{C.wuellerstorfi}$ record (blue) to the benthic LR04 stack[26] (red). (b) According depth/age relationship for the established age

model. Crosses indicate nannofossil biodatums and triangles indicate planktic foraminiferal biodatums (depths from ref. 24 and www.odsn.de).

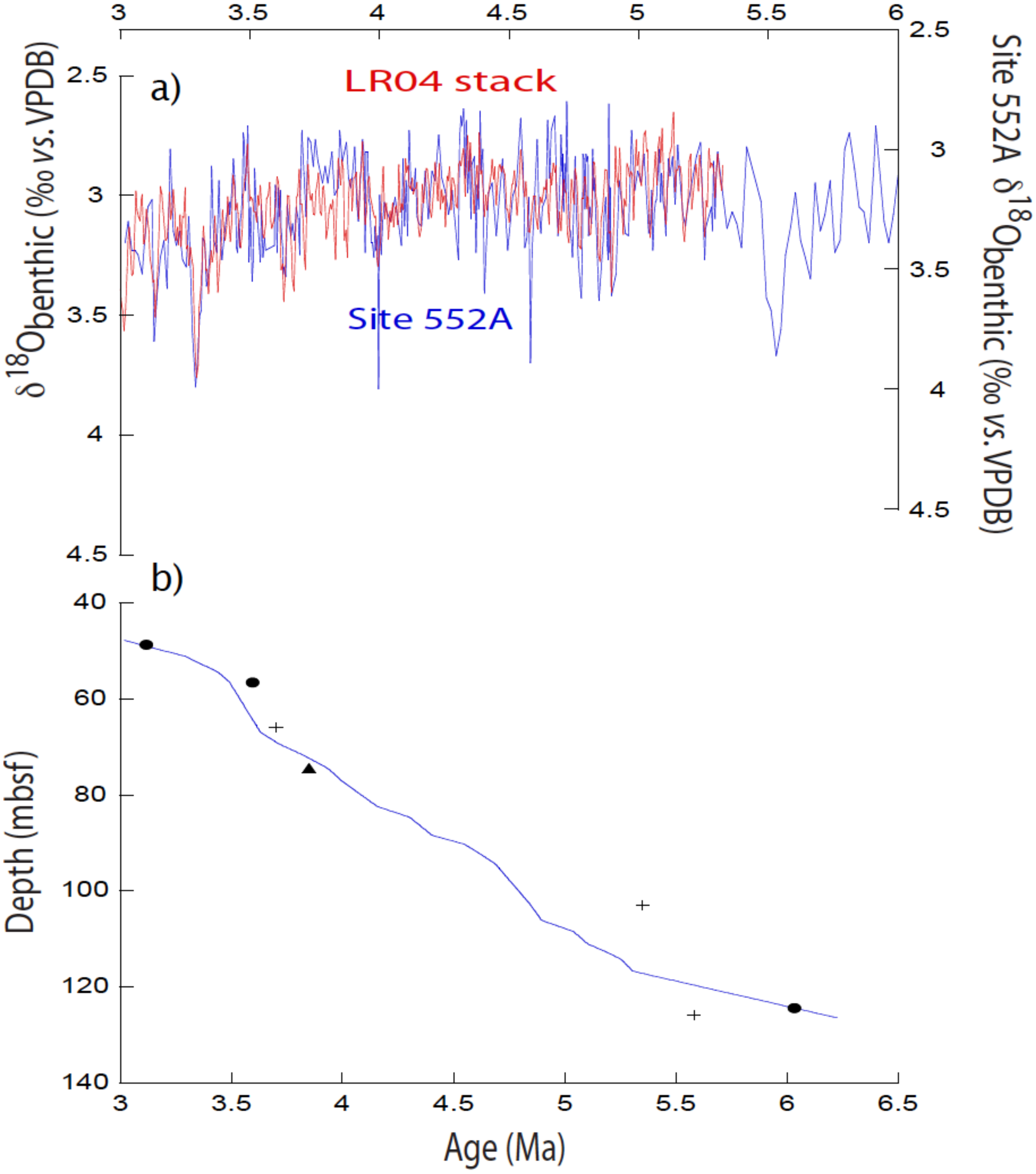

S-Fig. 3: Age model for Site 552A. (a) Tuning of the high resolution benthic $\delta^{18}O_{C.wuellerstorfi}$ record (blue line) to the global benthic reference stack LR04[26] (red line). (b) Depth/age relationship for Site 552A. Crosses (triangles) indicate nannofossil (foraminiferal) biodatums, and dots mark magnetostratigraphic reversal datums (depths were taken from refs. 28, 29, 30).

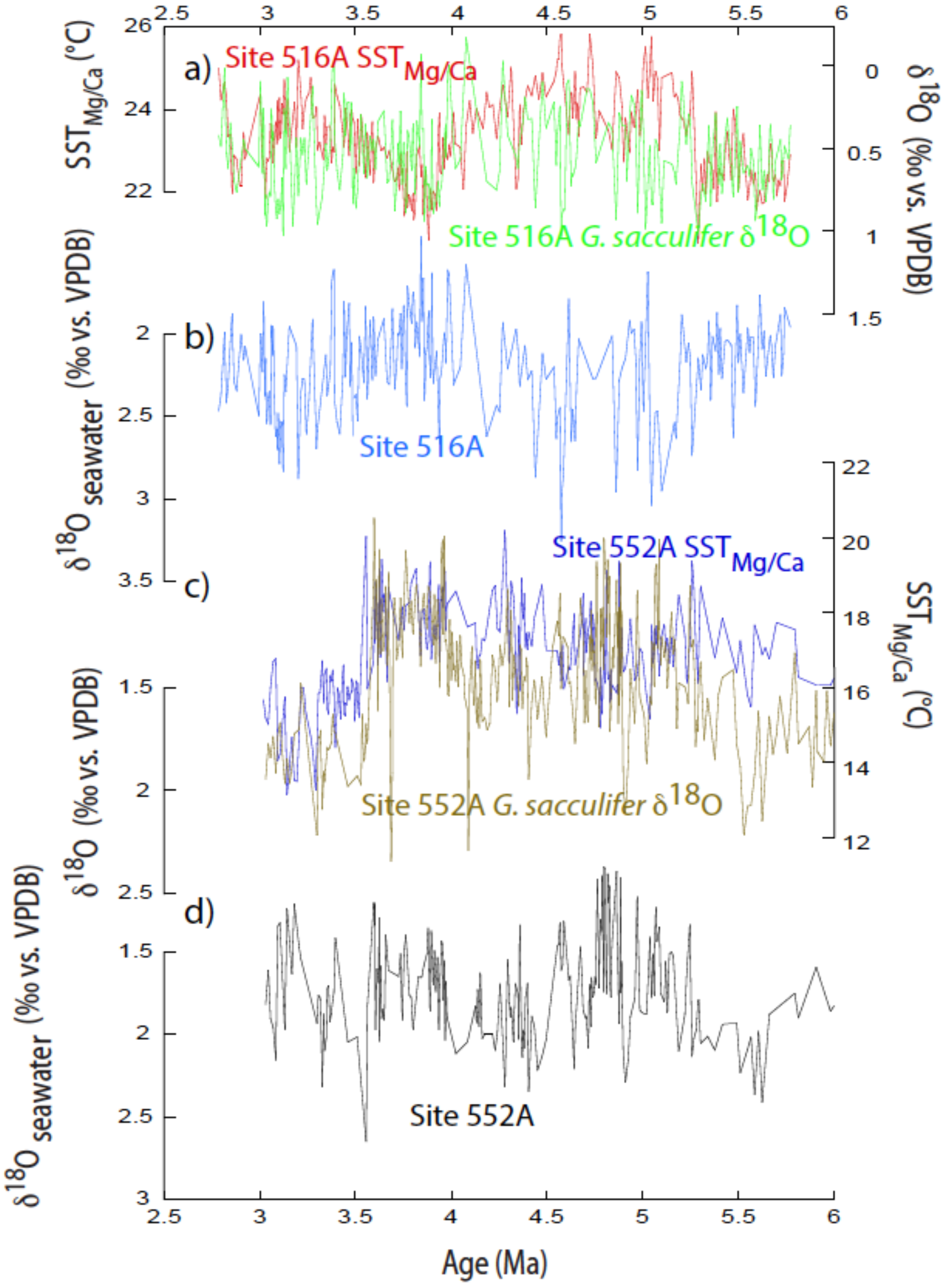

S-Fig. 4: Calculation of $\delta^{18}O_{seawater}$ of sites 516A and 552A. (a) *G. sacculifer* $SST_{Mg/Ca}$ (red) and *G. sacculifer* $\delta^{18}O$ (green) records of Site 516A were used to calculate the (b) $\delta^{18}O_{seawater}$ record of Site 516A (blue) applying the equation of ref.[32]. (c) Accordingly, *G. bulloides*

$SST_{Mg/Ca}$ (blue) and *G. bulloides* $\delta^{18}O$ (brown) records of Site 552A were used to calculate the (d) corresponding $\delta^{18}O_{seawater}$ record of Site 552A (black)[32].
